# The complete mitochondrial genome sequence of *Sinopoppia nigroflagella* Wei, 1997 (Hymenoptera: Tenthredinidae) reveals a new gene order

**DOI:** 10.1080/23802359.2021.1891989

**Published:** 2021-03-22

**Authors:** Siying Wan, Meicai Wei, Gengyun Niu

**Affiliations:** College of Life Sciences, Jiangxi Normal University, Nanchang, Jiangxi, PR China

**Keywords:** Mitogenome, *Sinopoppia*, gene rearrangement, phylogenetic analysis

## Abstract

The complete mitochondrial genome of *Sinopoppia nigroflagella* Wei, 1997 was sequenced and assembled. The circular genome is 15,940 bp long, with an A + T content of 80.33%, 37 genes, and a 658-bp control region. Specifically, *trnL1* was translocated into the MQI gene cluster, and the other tRNA cluster was arranged as ARENS1F. The two gene clusters were thus arranged as ML1QI and ARENS1F. The phylogenetic results indicated that *S. nigroflagella* forms a sister group with *Blennocampinae* and *Fenusinae*.

*Sinopoppia nigroflagella* is a peculiar sawfly species and its systematic position remains uncertain (Wei 1997). Wei and Nie (1998) placed *S. nigroflagella* into Caliroinae of Heterarthridae. Taeger et al. (2010) treated it as a member of Heterarthrinae of Tenthredinidae. Based on COI data *S. nigroflagella* seemed to be combined with the *Dimorphopteryx* species of Tenthredininae (Unpublished data). The phylogenetic position of *S. nigroflagella* remains to be determined. In this study, we sequenced the mitochondrial genome of *S. nigroflagella* to determine its phylogenetic position.

Specimens were collected from the Xinting, Jiulong, Lishui, Zhejiang, China (28.404 N, 119.838 E) on 31 March 2018 by Zejian Li, and identified by Meicai Wei. The specimen was deposited at the Asia Sawfly Museum, Nanchang (ASME) (Meicai Wei, weimc@126.com) under the voucher number CSCS-Hym-MC0073. Genomic DNA was sequenced using the high-throughput Illumina Hiseq 4000 platform, yielding a total of 90,672,018 raw reads (SRR13487075). DNA sequences were assembled using MitoZ (Meng et al. 2019) and Geneious Prime 2019.2.1 (https://www.geneious.com). Annotations were generated using the MITOS web server (Bernt et al. 2013) and revised where necessary.

Phylogenetic analysis was performed, including only hymenopteran taxa, to avoid possible effects of long-branch attraction. The nucleotide sequences of 13 protein-coding genes (PCGs) of 50 other Symphytan and two Apocritan species were aligned using the MAFFT method in the TranslatorX server (Abascal et al. 2010). The phylogenetic tree was constructed using IQTREE (Jana et al. 2016) with default parameters. Based on the previous experience (Malm and Nyman 2015), to avoid the long-branch attraction, the outer group was excluded. Thus, Xyelidae was used for rooting the tree.

The sequence yield by MitoZ was 16,730 bp in length and contained 37 genes with an incomplete control region. We used the sequence flanking the control region as a reference sequence. One of the flanking regions contained *trnM* (120 bp) and the other included part of the *rrnS* (110 bp) gene. The results of the two assemblies were consistently showing them as a control region 658 bp in length. We then verified the 15,940 bp long genome using * Endemyolia tibialis* (unpublished) as the reference sequence and the mean depth of coverage across the contig sequence was 1180.

Compared to the ancestral insect mitochondrial genome (Boore 1999), three rare rearrangement events were detected in the mitochondrial genome of *S. nigroflagella*. The *trnQ*(−) and *trnM*(+) genes were shuffled with *trnL1*(−) translocated downstream of *trnM*(+). The rearrangement of *trnL1*(−) was verified using *trnM*(+) and *trnQ*(−) as reference sequences, respectively. The last rearrangement event was *trnE*(+) remote inversion to the downstream region of *trnR*(+). The two gene clusters were thus arranged as ML1QI and ARENS1F.

The A + T content of the whole mt genome was 80.33% (43.05% A, 11.94% C, 7.73% G, and 37.28% T), indicating significant A + T bias. Six start codons for PCGs were used; ATA (*nad2*); ATT (*cox2*, *atp8*, *nad3*, and *nad5*); ATG (*cox1*, *atp6*, *cox3*, *nad4*, and *cob*); ATC (*nad4L*); TTG (*nad1*); GTG (*nad6*). All PCGs used TAA as a stop codon except for *nad2* (TA) and *nad4* (T). A total of 319 intergenic nucleotides were observed in the whole mitochondrial genome with the longest (38 bp) identified between the *rrnL* and *nad1* genes.

Phylogenetic analysis indicated that *S. nigroflagella* is a sister group of the lineage of *Blennocampinae and Fenusinae*. The clade of *S. nigroflagella* + (*Blennocampinae and Fenusinae*) forms a sister group of the clade combined by Tenthredininae and Allantinae. This study further clarified the phylogenetic position of *Sinopoppia* in the Tenthredinidae. However, considering the complexity of Tenthredinidae, dense sampling is essential to obtain a robust phylogeny (Figure 1).

**Figure 1.**
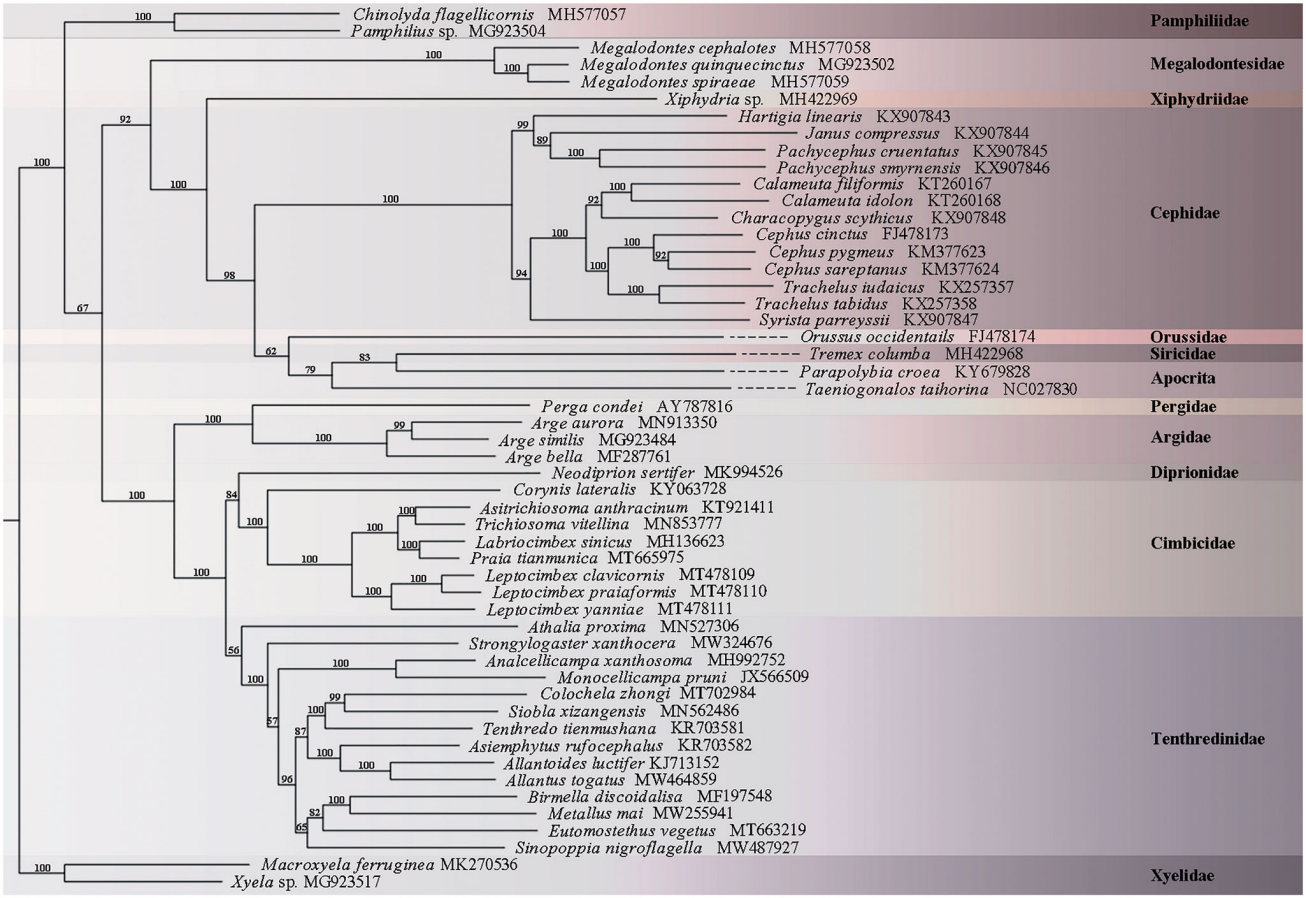
A maximum likelihood (ML) tree based on the combined data of the 13 protein-coding genes of 52 hymenopteran species.

## Data Availability

The genome sequence data that support the findings of this study are openly available in GenBank of NCBI at (https://www.ncbi.nlm.nih.gov) (https://www.ncbi.nlm.nih.gov/) under the accession number MW487927. The associated BioProject, SRA, and BioSample numbers are PRJNA692343, SRR13487075, and SAMN17320634, respectively. All related files had been uploaded to figshare (https://figshare.com/account/home#/projects/100472).
